# Gluteal transposition flap without donor site scar for closing a perineal defect after abdominoperineal resection

**DOI:** 10.1007/s10151-016-1552-1

**Published:** 2016-11-28

**Authors:** R. D. Blok, O. Lapid, W. A. Bemelman, P. J. Tanis

**Affiliations:** 1 Center for Experimental and Molecular Medicine, Academic Medical Center, University of Amsterdam, Amsterdam, The Netherlands; 2 Department of Surgery, Academic Medical Center, University of Amsterdam, Post box 22660, 1100 DD Amsterdam, The Netherlands; 3 Department of Plastic and Reconstructive Surgery, University of Amsterdam, Amsterdam, The Netherlands

## Introduction

Abdominoperineal resection (APR) is still associated with substantial morbidity related to the perineal wound [1]. Perineal wound problems are observed in up to 47% of patients, with secondary hernia formation in up to 26% [2, 3].

Obliterating the perineal dead space with well-vascularized tissues can promote wound healing after primary APR or can be used to treat secondary complications.

**Fig. 1:**
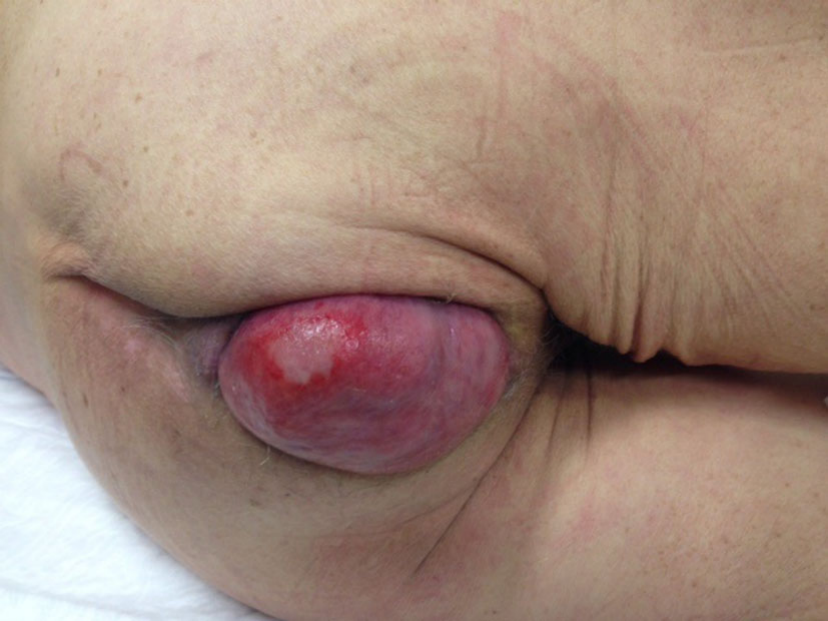
Protrusion of a small bowel loop through a granulating perineal wound after abdominoperineal resection

We present a case of emergency surgery in a patient presenting with small bowel herniation through an unhealed perineal wound (Fig. 1) 2 months after APR for pT3N0M0 rectal cancer. Following pelvic floor reconstruction with a biological mesh, the perineal soft tissue defect was closed using a unilateral semicircular gluteal perforator flap, which we named Luna flap, followed by midline closure of the skin.

## Surgical technique

**Fig. 2:**
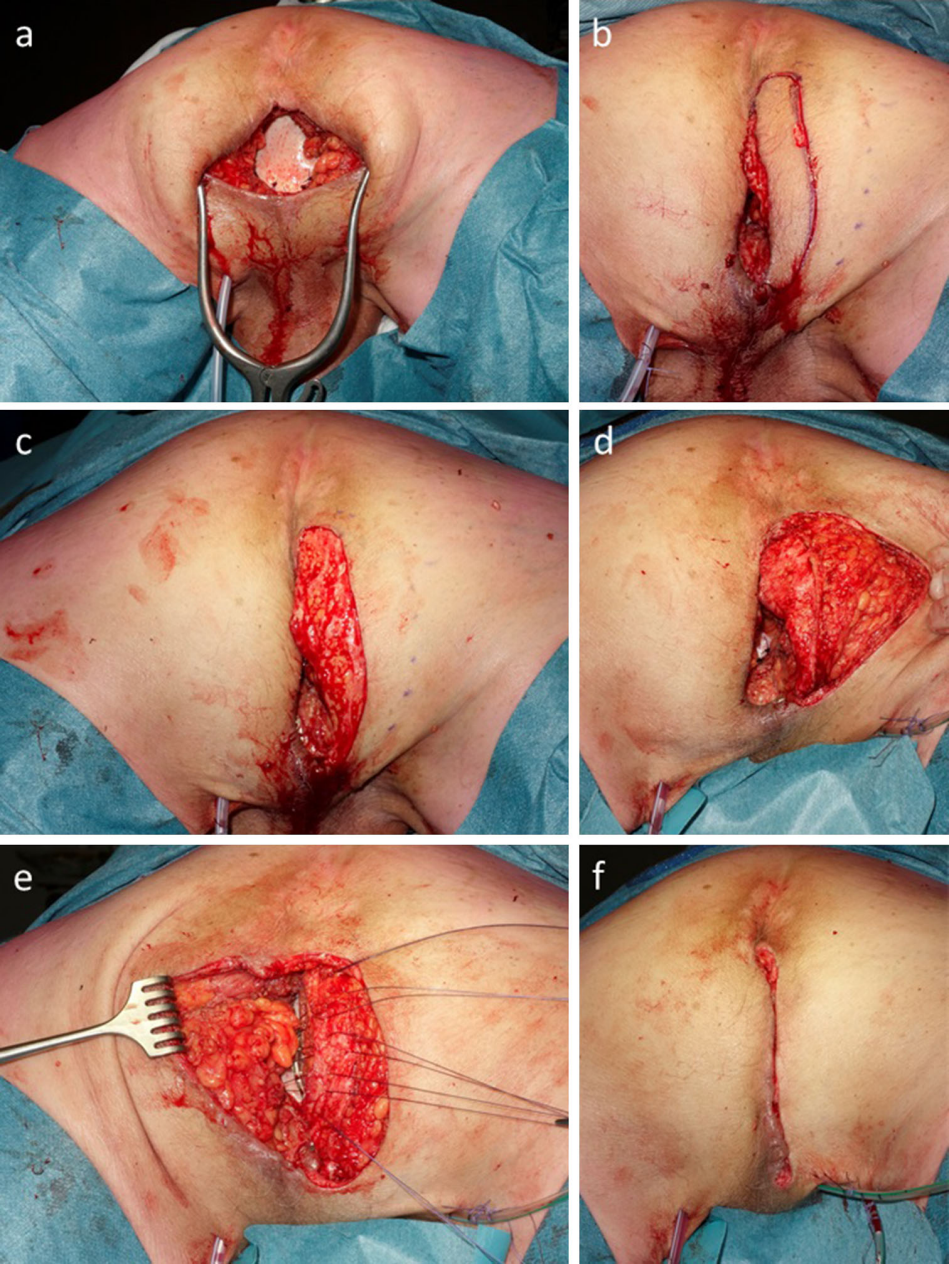
**a** Pelvic floor reconstruction with Strattice 6 × 10 cm with intra pelvic silicone drain, **b** incision of Luna flap, **c** deepithelialization, **d** transection of subcutaneous fat **e** deep fixation of the flap over a CH10 Redon drain, **f** closure of the midline over a second CH10 Redon drain

The patient was placed in the prone position. Granulation and fibrotic tissue was excised with detachment and repositioning of the small bowel loop. The pelvic floor was reconstructed by stitching an acellular biological mesh (Strattice™, 6 × 10 cm) to the sacrococcygeal ligaments, remnants of the levator muscle and transverse perineal muscles with interrupted Monoplus 2/0 sutures. A silicone drain was inserted in the pelvic cavity behind the mesh, because there was still a purulent discharge from the perineal wound. Next, a shallow semicircular incision was made in the right gluteal skin with a maximum distance of about 3 cm from the adjacent perineal defect, including at least one perforator of the gluteal artery as identified by Doppler imaging (Fig. 2). The Luna-shaped skin island was deepithelialized. The subcutaneous fat was transected lateral from the perforator down to the gluteal fascia. Afterward, the subcutaneous flap was placed onto the biomesh and fixed with Novosyn 3/0 sutures, completely obliterating the remaining dead space. A vacuum drain was placed between the mesh and the flap. The subcutaneous tissue on both sides of the wound was slightly mobilized from the gluteal fascia and closed in the midline over a second vacuum drain, followed by intracutaneous closure of the skin in the midline.

**Fig. 3:**
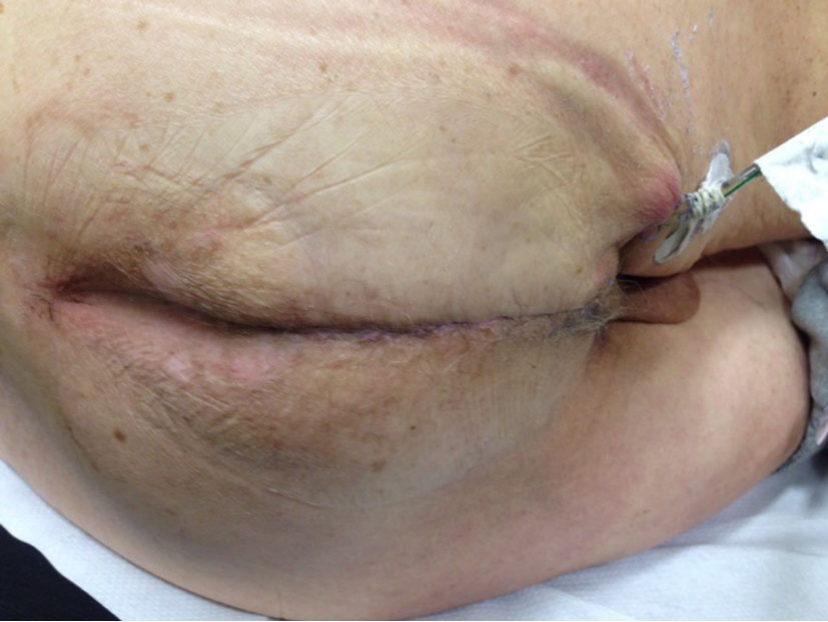
Healed perineal wound at 3 weeks

Postoperatively, continuous purulent discharge from the silicone drain in the pelvic cavity required twice-daily irrigation with saline solutions. After 7 days, the patient was discharged with the silicone and deep vacuum drains still in situ. The patient was fully mobilized after 2 days and was allowed to sit after 10 days. The silicone drain fell out after 2 weeks, but clinical examination at the outpatient clinic at 3 weeks showed a well-healed perineal wound (Fig. 3) and the remaining vacuum drain was removed. Follow-up after 6 weeks is still uneventful.

## Discussion

A small bowel herniation in an unhealed perineal defect after APR demanded emergency surgery. A biomesh was used to reconstruct the pelvic floor. The dead space above this mesh was covered with a gluteal perforator flap. No additional scars were required.

Because a subcutaneous transposition flap probably does not add any strength to the pelvic floor, a biological mesh was chosen for reconstruction in a contaminated environment. It is of great importance to sufficiently cover the mesh with soft tissue to prevent seroma and abscess formation below the mesh and to promote mesh ingrowth. Currently, there are several options for filling a perineal defect, but all are associated with the risks of donor- and recipient-site morbidity [4, 5]. The Luna flap as described in the present report seems to be a promising modification of the VY fasciocutaneous gluteal transposition flap for complete filling of the dead space in relatively small perineal defects, without additional scars, with an early return to normal activity and only limited increase in operative time.
